# The Goals and Effects of Music Listening and Their Relationship to the Strength of Music Preference

**DOI:** 10.1371/journal.pone.0151634

**Published:** 2016-03-17

**Authors:** Thomas Schäfer

**Affiliations:** Department of Psychology, Chemnitz University of Technology, Chemnitz, Germany; University of Marburg, GERMANY

## Abstract

Individual differences in the strength of music preference are among the most intricate psychological phenomena. While one person gets by very well without music, another person needs to listen to music every day and spends a lot of temporal and financial resources on listening to music, attending concerts, or buying concert tickets. Where do these differences come from? The hypothesis presented in this article is that the strength of music preference is mainly informed by the functions that music fulfills in people’s lives (e.g., to regulate emotions, moods, or physiological arousal; to promote self-awareness; to foster social relatedness). Data were collected with a diary study, in which 121 respondents documented the goals they tried to attain and the effects that actually occurred for up to 5 music-listening episodes per day for 10 successive days. As expected, listeners reporting more intense experience of the functional use of music in the past (1) had a stronger intention to listen to music to attain specific goals in specific situations and (2) showed a larger overall strength of music preference. It is concluded that the functional effectiveness of music listening should be incorporated in existing models and frameworks of music preference to produce better predictions of interindividual differences in the strength of music preference. The predictability of musical style/genre preferences is also discussed with regard to the present results.

## Introduction

Music is of great importance in the lives of most people all over the world. In the Western hemisphere, people deliberately listen to about 18 hours of music per week [1]. With the availability of music on the Internet and the mobility of access to music via portable devices, music has become a ubiquitous companion of many individuals, especially adolescents. It goes without saying that this overwhelming presence of music in the lives of so many people will affect their way of life profoundly—how they feel, how they perceive, how they think, and how they behave. Many of these effects are already well understood and it is known that many of them significantly depend on how much the music is liked or preferred [2,3,4]. It is therefore highly important to understand the causes of music preference (MP) in detail.

Individual differences in MP are still among the most mysterious phenomena in psychology. Everyone easily admits liking particular musical styles, tunes, artists, or performers more than others. It is also easily recognized that other individuals will have different preferences. This aspect of MP has been called style/genre preference [5]. Moreover, people differ in the degree to which they are involved in listening to music, that is, in the degree to which they enjoy and love both music in general and what they call their “favorite” music. This aspect of MP has been called the *strength* of preference [6].

Where do these interindividual differences in style/genre preferences and strength of MP come from? Past studies have revealed some knowledge about the determinants of style/genre preferences but little in the way of determinants of the strength of MP. The strength of MP—which has rarely been the focus of past studies on music listening—is central to the fascinating question of why humans listen to music at all. Asking only what style of music a person prefers over another teaches us nothing about why that person actually turns on the radio or listens to music at all. Asking instead about the strength of MP reveals much about the actual engagement and involvement in listening to music [7]. A strong preference indicates that a person is highly involved in listening to music and actively seeks music by, say, turning on the radio, buying music, or attending concerts. The investment of temporal and financial resources indicates that listening to music must exhibit some kind of usefulness that manifests itself in the strength of MP [6,8,9]. The aim of the present work was to analyze the influence of the functionality of music listening—in terms of its goals and effects—on the strength of MP. The results also have potential implications for the prediction of style/genre preferences, which is why the state of research of both the type and the strength of MP is discussed first.

### Predicting Musical Style/Genre Preferences

How can individual differences in musical style/genre preferences be predicted? Age, sex, some personality characteristics, social, and sociocultural variables have turned out to be the most promising variables for making good predictions. Knowledge about the influence of age on MP is the most conclusive. People who maintain a certain interest in music over their lifetime typically prefer increasingly complex music [2], whereas people who hardly engage in music typically prefer easily comprehensible music when they get older [10] or music that was popular when they were in their adolescence [10,11]. Sex does not appear to have a strong influence on MP apart from some tendencies, such as that males prefer harder or more energetic music styles than females [2]. Research on personality correlates of MP has yielded many small to medium-sized correlations, but these have not been consistent across studies. Some of the more prominent findings refer to personality traits such as sensation seeking and the Big Five. Sensation seekers—individuals with a heightened need for novel, complex, and risky experiences—tend to prefer more arousing and activating music [12,13,14]. The Big Five personality traits have been shown to correlate with very general musical style features. For instance, openness to experience is correlated with a preference for “intense and rebellious” and “reflective and complex” music while extraversion is correlated with a preference for “upbeat and conventional” and “energetic and rhythmic” music [5,15,16]. There are many social influences on the emergence and development of MP. Parents and teachers can bring children in contact with certain types of music or encourage them to engage in musical activities such as learning an instrument, which is associated with certain types of music, as well [17]. Later in life, there is an increasing influence of peers on the consolidation or reshaping of preferences [18], mainly because music becomes a means to express social relatedness and define one’s identity [19,20]. Not least, on a very general level, MPs are informed by cultural traditions such as certain syntactical norms of music or certain affective associations and meanings [21]. In sum, there are many variables that have an influence on musical style/genre preferences, and altogether these can explain a certain part of the variance. Given the rather small correlations between these variables and MP, it seems, however, that the lion’s share of the variance remains unexplained.

### Predicting the Strength of Music Preference

Although the strength of MP has rarely been the focus of empirical studies, researchers have of course asked participants to report it; but this was done only to learn about which styles or genres listeners prefer. For instance, questionnaires such as the Short Test of Music Preference [5] ask participants how much they like different musical styles, but these quantitative measures are usually used only to identify which musical styles/genres/characteristics a listener likes most, while the absolute magnitude and interindividual differences in these measures are usually not of interest. The question of why different listeners exhibit different levels of mean strength of preference has been widely neglected. In fact, even the strength of preference for people’s favorite music varies considerably [6].

The same variables that predict musical style/genre preferences can be used to predict the strength of MP. But in this case, too, most of the variance remains unexplained. In recent years, however, the *functionality of music listening* has been discussed as a variable central to the understanding of the strength of preference. Functionality refers to the deliberate use of music to attain specific goals in specific situations, such as to elicit positive emotions when in a negative mood, to strengthen social relationships, or to manage one’s physiological arousal. Behne [8], for instance, argued that the intensity of what he called *Musikerleben* should be expressible in terms of the functions that music fulfills for the listener. Regarding the potential functionality of music, he further suggested that “individual characteristics of music appreciation must be interpreted in the context of individual history as individual ways of coping with life” ([22], p. 154). Lehmann [23] also reasoned that the functionality of music listening might be the most important reason for the development of MP. His situation–functions–preference model states that listeners focus on intended effects (goals) of music listening that emerge from the actual situation and the listener’s learning history; since a specific music is capable of fulfilling the listener’s expectations, the listener is likely to establish a habit of listening to this music again in the future. When investigating the development of MP over the life span, Bonneville-Roussy et al. ([10], p. 705) argued that “individuals use music to satisfy and reinforce basic psychological needs.” Further, the uses-and-gratifications approach—introduced into music cognition, for instance, by Arnett [19] and Larson [20]—holds that people actively used media such as music to attain specific goals.

Although the outlined approaches are compelling and promising, only a handful of empirical studies have investigated the functional aspect of music listening and its influence on MP [24]. The present research was intended to clarify the relationship between the functionality of music listening and the strength of MP.

### Using the Functions of Music to Predict the Strength of MP

Building on previous studies [6,25,26,27] and taking into account the large amount of knowledge on the functions of music listening [28] and the theoretical approaches of Behne [8] and Lehmann [23], the following theoretical framework was derived: Over time, individuals learn that listening to music can help them attain specific goals in specific situations (*past functional experiences*). That is, music listening can be a functional behavior. Past functional experiences gradually manifest in specific intended effects of music listening (*goals*). The more listening to music is able to fulfill specific functions consistently—so that the intended *effects* really occur—the more it will become a habit, and there will be an increase in the involvement in and enjoyment of music listening or, in other words, in the *strength of MP*. If listening to music does not help the listener attain goals it will not get reinforced and preference for the music will remain weak.

**Fig 1:**
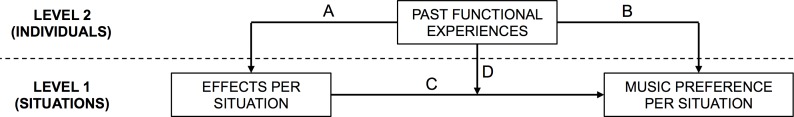
Multilevel model of the proposed relationships between past functional experiences, the effects of music listening, and the strength of music preference.

Using this framework, two questions were explored and four specific predictions were tested (see also Fig 1). The two questions refer to a quantification of the significance of goals and effects: (1) To what extent do listeners intend to attain specific effects in everyday music-listening situations? These are the goals of music listening. (2) To what degree can music really help the listener attain these goals? These are the effects of music listening. The four predictions refer to the above learning hypothesis: In actual everyday situations, (1) the strength of a listener’s past functional experiences can predict the degree of goal attainment, that is, the strength of the effects of music listening (effects); (2) the strength of a listener’s past functional experiences can predict the strength of preference for the music that is listened to; (3) the strength of the effects of music listening can predict the strength of MP; and (4) the strength of a listener’s past functional experiences can predict the strength of the association between the effects of music listening and the strength of MP. Prediction 4 refers directly to the influence of individual differences, but this requires explanation. Imagine two people listening to music in different situations. For one person, there might be a strong correlation between goal attainment and MP: When the music can help fulfill specific goals, preference for this music is high; when the music fails to fulfill the same goals for some reason, preference will be low. For the other person, in contrast, there might be a weak correlation between goal attainment and MP: Whether the goals of this listener are attained through the music or not is barely reflected in the strength of preference for this music. Given that this variation between different people really exists, the present approach allows a straightforward explanation: Individuals who have had consistently positive experiences with the functionality of music in the past will know which music to listen to in order to attain specific goals in specific situations. Consequently, they will exhibit a strong association between goal attainment and MP. In contrast, individuals who have rarely had such positive experiences in the past will exhibit a comparably weak association between goal attainment and MP.

## Method

### Participants

In total, 121 volunteers participated in the study (aged 18 to 66 years, *M* = 28.5 years, *SD* = 11.5; 72 female, 49 male). Most of the participants (73%) were students; in terms of musical experience, 79% played an instrument and 33% sang, 21% were full-time professional musicians, and 16% were music students. They were recruited via e-mail or personal request.

### Ethical Approval

The study was performed in accordance with relevant institutional and national guidelines and regulations [22,29] and with the principles expressed in the Declaration of Helsinki. Formal approval of the study by the Ethics Committee of Chemnitz University of Technology was not mandatory, since the study adhered to all the required regulations. Anonymity of participants and confidentiality of their data were ensured. They were informed about the objectives and the procedure of the survey as well as about their right to withdraw from the study at any time without adducing reasons and without any negative consequences. All participants gave their informed consent online in accordance with the guidelines of the Ethics Committee of Chemnitz University of Technology, by ticking a checkbox.

### Procedure

Over 10 successive days, respondents were asked to be mindful of situations where they listened to music during the day. They were advised to carry a memo pad to make notes about these situations, if possible. To minimize retrospective bias, they were asked to use their notes when completing the questionnaire in the evening. Each evening—at a time convenient to the participants—they answered a detailed online questionnaire (diary). For each music listening situation per day, they were asked to remember the goals and effects of music listening—always keeping in mind the three fundamental functions of music listening identified by Schäfer, Sedlmeier, Städtler, and Huron [28]: self-awareness (self-related cognitions and emotions), social relatedness (social bonding and affiliation), and arousal and mood regulation (background entertainment and diversion). The three functions were explained in detail and examples were given.

Regarding the goals, respondents were asked three questions: (1) How important was *self-awareness* for your intention to listen to music in that situation? (2) How important was *social relatedness* for your intention to listen to music in that situation? (3) How important was *arousal and mood regulation* for your intention to listen to music in that situation? Regarding the effects, respondents were asked (1) “How strongly did the function of *self-awareness* actually occur through listening to music?” (2) “How strongly did the function of *social relatedness* actually occur through listening to music?” (3) “How strongly did the function of *arousal and mood regulation* actually occur through listening to music?” All questions were answered on a scale of 1 (*not at all*) to 10 (*very much*). Regarding past functional experiences, respondents were asked, “How intensely have you used the same music in the past to attain specific goals?” This question was answered on a scale of 1 (*never before*) to 10 (*very often*). For each situation, respondents were also asked if they were free to decide about the music they listened to or if it was preselected music (such as a radio broadcast), if they were alone or with others, and for how long they listened to music. Finally, they were asked, “How much did you like the music you had listened to in that situation?” This question was answered on a scale of 1 (*not at all*) to 10 (*very much*).

For the sake of manageability, participants were asked to answer the diary questionnaire at fixed intervals, that is, every evening [30]. The questionnaire was limited to five situations per day [31].

## Results

### Analysis Strategy

First, data of the goals and effects of music listening were analyzed by computing the averages for the three fundamental functions across all participants and all listening situations. Mean differences between goals and effects were analyzed using *t* tests.

Second, the influence of the listener’s past functional experiences on the effects of music listening and the strength of MP was analyzed. For these analyses, data of the three fundamental functions of music listening were averaged. Because the variables involved in the predictions unfold on different levels of measurement (i.e., within and between individuals), hierarchic multilevel modeling was used to analyze the data. Level 1 contains the different music-listening situations of each participant. Level 2 contains the participants who can be characterized by the mean strength of their past functional experiences, their age, sex, and musical experience. The mean strength of past functional experiences was calculated by taking the average of the past functional experiences data from all music-listening situations for each participant.

Parameters were estimated using the software HLM [32]. Estimates were calculated for the following coefficients. In Model 1, the dependent variable was the strength of the effects of music listening. The intercept of this variable was predicted by past functional experiences (means as outcomes model; see Path A in Fig 1). In Model 2, the dependent variable was the strength of MP. The intercept of this variable was predicted by past functional experiences (means as outcomes model; see Path B in Fig 1). The variable itself was predicted by the effects of music listening (simple regression model; see Path C in Fig 1). Finally, the slope of the relationship between effects of music listening and the strength of MP was predicted by past functional experiences (slopes as outcomes; see Path D in Fig 1). In both models, the effects of the Level 2 predictor past functional experiences were controlled for other Level 2 variables that might affect the strength of MP: age, sex, and musical experience. Musical experience comprised four dummy-coded variables: playing an instrument (0 = no, 1 = yes), singing (0 = no, 1 = yes), being a music student (0 = no, 1 = yes), and being a full-time professional musician (0 = no, 1 = yes). For each predictor variable, the amount of variance this variable is able to explain in the dependent variable (Δσ^2^) was estimated by comparing the residual variance of the model without ($\sigma_{\varepsilon 0}^{2}$) this predictor and the residual variance of the model with ($\sigma_{\varepsilon }^{2}$) this predictor: ${\Delta \sigma }^{2}=1-\frac{\sigma_{\varepsilon }^{2}}{\sigma_{\varepsilon 0}^{2}}$. Model fit was estimated by comparing the conditional model (containing all predictors and controls) with the unconditional model, resulting in a difference in deviance, which in HLM is inferentially tested using a χ^2^ statistic.

### Goals and Effects of Music Listening

In total, participants reported 1,502 music-listening situations over the 10-day period. On average, they reported 1.4 situations per day (*SD* = 0.67) with a mean duration of 81.4 min (*SD* = 86.3). In 67% of the situations, they were able to select their own music. In most of the situations (60%), participants were alone; in 15% of the situations, they were with their significant other, and in 13% of the situations, they were with friends. Music listening occurred in very different settings: in a car or on a train, bus, or bike (22%); while doing homework (21%), concentrating on the music (14%), or relaxing (10%); during meals (8%); while socializing with friends (6%), exercising (4%), watching television or gaming (2%), or attending church (1%). The mean preference for the music that was listened to was 7.8 (*SD* = 2.2). Looking only at the situations where respondents could select the music on their own, the preference was even higher, *M* = 8.5 (*SD* = 1.7).

**Fig 2:**
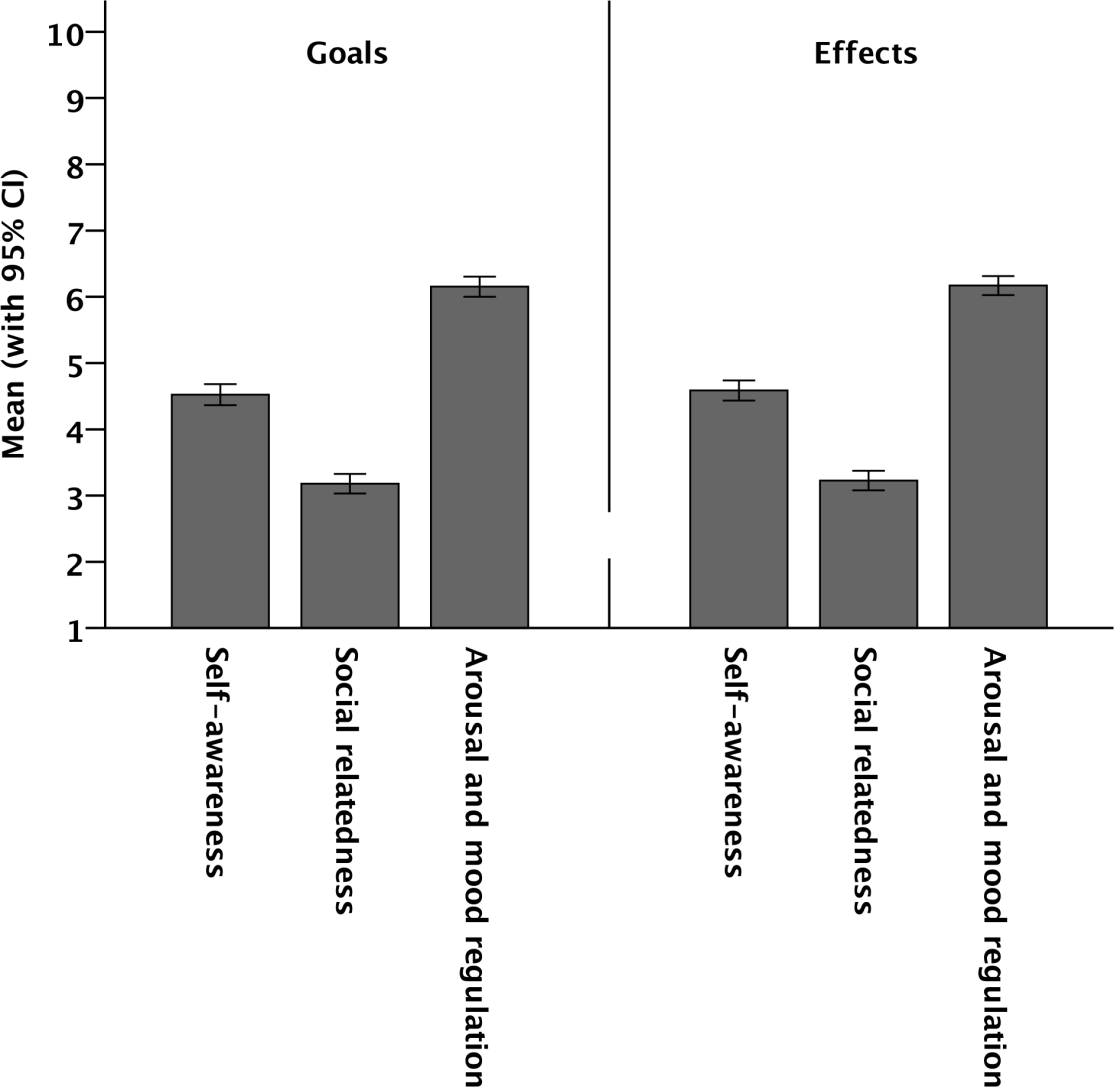
Mean intensity of goals and effects of music listening, calculated from 1,502 everyday music-listening situations.

The mean goals and effects of music listening over all situations are shown in Fig 2. (For the complete dataset of the study, see S1 and S2 Tables) The data reveal two main results. First, there were no mean differences between the goals and effects. Music listening appears to lead to specific effects to about the same degree as these effects were intended or desired. There were no substantial differences (self-awareness: *t* = 1.59, *p* = .11, *d* = .02; social relatedness: *t* = 1.53, *p* = .13, *d* = .02; arousal and mood regulation: *t* = 0.41, *p* = .68, *d* = .01). Second, the patterns of goals (self-awareness: *M* = 4.5, *SD* = 3.1; social relatedness: *M* = 3.2, *SD* = 2.9; arousal and mood regulation: *M* = 6.2, *SD* = 3.0) and effects (*M* = 4.6, *SD* = 3.0; social relatedness: *M* = 3.2, *SD* = 2.9; arousal and mood regulation: *M* = 6.2, *SD* = 2.8) remarkably mirror the pattern of the importance of the three fundamental functions of music listening found by Schäfer et al. [28]: Arousal and mood regulation emerges as the most important function, followed by self-awareness and social relationships.

### The Functionality of Music Listening and Its Influence on the Effects of Music Listening and the Strength of Music Preference

The hypothesis was that people have acquired their MP because they learned in the past that specific types of music helped them attain certain goals. The extent of such a positive history of learning was termed past functional experiences. Specifically, the following predictions were tested (see Fig 1): (1) The strength of past functional experiences can predict the strength of the effects of music listening (Path A); (2) the strength of past functional experiences can predict the mean strength of MP (Path B); (3) the strength of the effects of music listening can predict the strength of MP (Path C); and (4) the strength of past functional experiences can predict the strength of the relationship between the effects of music listening and the strength of MP over different situations (Path D). Predictions 1 and 2 directly derive from the idea of individual learning histories. In contrast, Predictions 3 and 4 also incorporate a current effect: In actual situations, the effects of music listening can predict the strength of preference for that music—and this relationship is stronger for individuals having had strong functional experiences in the past. This is an ultimate test of the central assumption of the present work: Given that there is variation in the correlation between the effects and MP, it was predicted that the listener’s learning history can explain it.

#### Past Functional Experiences and Effects of Music Listening

Because the functionality of music listening and its influence on MP are described as a learning process that unfolds over time, it was predicted that higher values in past functional experiences would result in higher values in the mean effects of music listening (see Path A in Fig 1). The data validated this prediction (see Table 1). The degree of music’s potential to help the listener attain specific goals in specific situations increases with the overall strength of positive functional experiences with music in the past. This is a novel finding indicating that the effects of music listening may have emerged through a learning history.

**Table 1:** HLM estimates of Model 1. Dependent variable: effects of music listening.

Level 2 predictors of the intercept (χ^2^ = 3.8, *p* = .048)^a^	*b*	*SE*	*t*	*p*	*df*	Δσ^2^
Past functional experiences	0.23	0.05	4.6	< .001	113	14.8
Control: age	-0.01	0.01	-0.7	.47	113	
Control: sex	0.26	0.19	1.4	.17	113	
Control: singing	0.25	0.21	1.2	.24	113	
Control: instrument	-0.07	0.18	-0.4	.70	113	
Control: music studies	0.44	0.26	1.7	.09	113	
Control: musician	-0.26	0.26	-1.0	.30	113	

*Note*. The *b* statistic is the unstandardized regression coefficient. Δσ^2^ is the amount of variance in the criterion the predictor is able to explain. The χ^2^ statistic is calculated from the difference between the deviance of the unconditional model and the deviance of the conditional model.

^*a*^See Path A in Fig 1.

#### Past Functional Experiences and the Strength of Music Preference

The same argument applies to the development of MP. If the functionality of music listening determines MP, higher values in past functional experiences should result in higher values in the mean strength of preference (see Path B in Fig 1). The data validated this prediction (see Table 2, Part A). The strength of preference for music listened to in specific situations increases with the overall strength of positive functional experiences with music in the past. This finding again illustrates that MP is a result of a learning history of functional experiences in the past.

**Table 2:** HLM estimates of Model 2. Dependent variable: strength of music preference.

Predictor	*b*	*SE*	*t*	*p*	*df*	Δσ^2^
Part A: Level 2 predictors of the intercept (χ^2^ = 37.1, *p* < .001)^a^						
Past functional experiences	0.42	0.04	9.4	< .001	113	49.1
Control: age	-0.01	0.01	-1.5	.14	113	
Control: sex	0.09	0.18	0.5	.63	113	
Control: singing	0.22	0.22	1.0	.32	113	
Control: instrument	0.01	0.10	0.1	.92	113	
Control: music studies	-0.16	0.23	-0.7	.49	113	
Control: musician	0.37	0.22	1.7	.10	113	
Part B: Level 1 predictor (χ^2^ = 277.8, *p* < .001)^b^						
Effects of music listening	0.58	0.05	12.0	< .001	113	20.5
Part C: Level 2 predictors of the slope of the relationship between the effects of music listening and the strength of music preference (χ^2^ = 257.5, *p* < .001)^c^						
Past functional experiences	0.02	0.03	0.7	.51	113	0.0
Control: age	0.00	0.05	0.2	.81	113	
Control: sex	0.01	0.10	0.1	.92	113	
Control: singing	-0.10	0.10	-1.0	.30	113	
Control: instrument	0.23	0.10	2.4	.02	113	
Control: music studies	-0.08	0.11	-0.7	.49	113	
Control: musician	0.02	0.10	0.2	.84	113	

*Note*. The *b* statistic is the unstandardized regression coefficient. Δσ^2^ is the amount of variance in the criterion the predictor is able to explain. The χ^2^ statistics are calculated from the difference between the deviance of the unconditional model and the deviance of the conditional model.

^a^See Path B in Fig 1.

^b^See Path C in Fig 1.

^c^See Path D in Fig 1.

#### Effects of Music Listening and the Strength of Music Preference

Calculated over all participants and situations, there was a strong relationship between the effects of music listening and the strength of MP (see Table 2, Part B). That is, the strength of MP is a function of the music’s potential to help the listener attain specific goals (Path C in Fig 1). This result ties in with previous findings about the important role the functions of music listening play in the prediction of MP [6, 24,25,26,27].

#### Past Functional Experiences and the Relationship Between the Effects of Music Listening and the Strength of Music Preference

Probably the most interesting prediction was that past functional experiences should be predictive of the strength of the relationship between the strength of the effects of music listening and the strength of MP in specific situations (see Path D in Fig 1). However, this prediction was not validated by the data (see Table 2, Part C). Although in this model the slope of the relationship between the effects of music listening and the strength of MP exhibited a significant amount of variance (*p* < .001), the strength of past functional experiences was not a significant predictor of this variance. Notably, the control variable playing an instrument turned out to have a significant influence on the relationship: Participants who played an instrument exhibited a stronger association between the effects of music listening and the strength of MP across different situations.

## Discussion

### Summary of the Results

The present study revealed that music listeners try to attain different goals to different degrees: Goals related to self-awareness and arousal and mood regulation seem to be quite important whereas goals related to social relatedness turned out to be less important. When the same listeners are asked how effective listening to music actually was in attaining their goals the same pattern emerged: Goals related to self-awareness and arousal and mood regulation were attainable whereas goals related to social relatedness not as much. There were no mean differences between the intended goals and the attained effects.

It was hypothesized that listeners who had had extensive experience with the effectiveness of music listening in the past would profit more from listening to music in specific situations, exhibit a stronger preference for the music they listen to in these situations, and also show a stronger correlation between the strength of music’s effectiveness and the strength of MP across many situations. Data supported the first two predictions but not the last. Thus, past functional experiences with music are a significant predictor of both the strength of music’s effectiveness in helping listeners attain situation-specific goals and the strength of preference for the music listened to in these situations. The results of the present study are discussed in detail below, followed by a discussion of potential limitations.

### Goals and Effects of Music Listening

The unlimited availability of music makes it easy for people to select whatever music they want in any situation. As North and Hargreaves ([2], p. 139) put it: “It would be surprising if people therefore did not use specific pieces of music to achieve very specific ends in very specific circumstances.” Many previous studies have analyzed people’s reasons for listening to music, yielding a multitude of different answers [28]. However, studies about the relative importance of different goals that listeners try to attain when turning on their radio or iPod are still rare. It was assumed that, like any other behavior, listening to music can be best understood when considered in terms of its usefulness. The first aim of the present research was to quantify the significance of goals and effects and answer two exploratory questions: (1) How significant are intended effects (goals) in everyday music-listening situations and (2) to what degree can music really help people attain these goals (effects)? It turned out that the strength of respondents’ intention to attain three goals mirrored the results of previous research [28]: The regulation of physiological arousal and moods appears to be the most important intention for turning on music, followed, in order of importance, by the aspiration for self-awareness and social relatedness. A second result was that the same pattern appears for the actual effects of listening to music. To conclude, the results suggest that the pattern of intended functions (goals) mirrors the pattern of actual effects. As was discussed by Schäfer et al. [28], these results also demonstrate that listeners appear to highly value a rather private use of music (self-awareness and well-being) whereas social motives (social relationships and relatedness) do not seem to be that important. When investigating musical engagement, Bonneville-Roussy et al. [10] arrived at the same conclusion.

### The Functionality of Music Listening and Its Influence on Music Preference

The second aim was to test specific predictions derived from the learning hypothesis. Results show that, first, listeners who had had more positive functional experiences with music in the past were more likely to attain their goals when they turned on their music. Second, listeners who had had more positive functional experiences with music in the past exhibited higher values in their preference for the music they listen to in general. Third, against the expectation, listeners with different strengths of functional experiences with music in the past did not differ in the strength of the correlation they showed between the effectiveness of music and the strength of their preference for that music across different situations. Instead, interindividual variation in the strength of this correlation was significantly influenced by whether participants had learned to play an instrument. Apparently, participants who played an instrument distinguished more between functional music and less functional music. It remains a task for future research to identify further variables that are responsible for the variation in the strength of the correlation between music’s effectiveness and the strength of MP.

To conclude, although the results do not fully support the theoretically derived hypotheses, they demonstrate that MP is determined partly by the functionality of music listening; it emerges and develops through a learning process where positive functional experiences give rise to new preferences or strengthen existing ones for specific songs or styles, and missing or negative experiences prevent the formation of new preferences or weaken existing preferences.

How can these new insights inform the development of more elaborate models of MP? Let us again distinguish between the strength and the type of MP. Previous research on differences in the strength of MP is rare. Schäfer and Sedlmeier [6] demonstrated that the strength of listeners’ preference for their own “favorite music” varies considerably and the intensity of the functional use of music can explain a large part of that variance. The present study ties in with those results, revealing that much of the variance of the strength of MP can be explained through the degree of goal attainment (i.e., the effects of music listening). Thus, models aiming to explain interindividual differences in the strength of MP should incorporate the strength of functional experience listeners have had with music in the past. These functional experiences may be the most reliable variable with which to predict the strength of preference for music in general. In other words, how much somebody is involved in music-listening activities overall very much depends on the intensity of positive outcomes through music-listening activities in the past. This finding may come as no surprise but music psychologists have not yet incorporated it in their models of MP. The empirical data collected in the current study suggest that they should do so.

The present results can also help refine models of musical style/genre preferences. Existing models [33,34] usually start “too late;” they begin with a given piece or style of music for which a preference decision is to be made. Thus, these models do not explain why listeners actually turn on their radio or iPod or why they select a specific style or tune. The present results suggest a straightforward answer to these questions: Listeners turn on music because they have specific situation-dependent goals they want to attain. They have the notion that the music can help them attain their goals, and they select a specific musical style or tune they know can make this happen. The present data suggest that a listener’s functional learning history with a musical piece or style is a very good predictor of whether it will be preferred. This new variable should be incorporated in existing models, for instance, as a new subfactor of the “characteristics of the listener.”

Of course, this conclusion leads to another two questions: (1) Given that listeners acquire their MP based on functional experiences with music, when in life does this happen? (2) Is it possible to predict what specific musical styles or tunes an individual will prefer based on these new results? The first question can be answered easily. A great many studies have shown that children do not have fixed MPs; instead they seem to be open to many different musical variations and styles—a phenomenon that has been dubbed open-earedness [35,36]. It turns out that children lose their open-earedness and start establishing concrete MPs around the age of 7–10 years. Some scholars have also conjectured that in the following years young people are confronted with issues of identity, personal relationships, love, and other concerns [19,37,38,39]. Of the many possible ways young people can cope with these issues, listening to music might be one of the most common, at least in the Western world. Other possibilities might be doing sports, meeting friends, or engaging in a hobby. Which of these possibilities is used likely depends on the social, cultural, and economic circumstances. Those who regularly use music as a means to attain some of their goals have a good chance of having positive experiences and learning that music can be used in a functional way. These are the people who will likely develop a strong preference for music in general and specific preferences for musical styles or tunes they have used to attain their goals. These acquired preferences are, of course, malleable to some degree; people can add new functional experiences with new musical styles or pieces throughout their life and develop new preferences. Yet, anyone who has had few or no functional experiences with music in the past is unlikely to develop a strong preference later on.

Let us now turn to the second question. Are the specific musical styles or pieces somebody prefers predictable? Obviously, for this to be possible, it is necessary to predict which styles or pieces have so far proved useful in an individual’s lifetime. I tend to deem such prediction an almost unsolvable challenge. As just mentioned, there are social, cultural, and economic circumstances that influence what kind of activity besides music a person will choose to cope with life’s highs and lows and what kind of music a person will come across that gets the chance to prove useful. To make a halfway reliable prediction about that kind of music, one would have to identify and measure a host of variables. Image a young girl in her teens living with her parents, who are not musicians, in a rural area. The closest town with a discotheque is a couple of miles away. At the age of 14 years, she starts going there every Saturday night with some of her friends from school. The discotheque was founded by a former bluegrass band and so bluegrass is what is played there most of the time. The girl likes this place because she can hang out with her friends, meet new people, and have a really good time. One day she falls in love with a boy she meets there—a love story she will never forget. From that point on, she can listen to pieces of music from that time to reminisce and revive the pleasant feelings she had. The discotheque and the music have accompanied and facilitated some of her best experiences in life. Framed in terms of the central hypothesis of the present article, the music proved useful once, and it still does. Most likely, the girl in our story will have developed a preference for bluegrass music, which she is likely to keep throughout her life.

The point I am trying to make with this anecdote is that we can imagine an infinite number of such stories. There are an infinite number of ways social, cultural, and economic variables can interact and eventually bring an individual in contact with a certain style of music that can eventually prove useful. In terms of psychological theorizing, most of these influences are exogenous or can simply be considered random. Of course, as mentioned in the Introduction, research has already revealed a handful of variables that can be used to make a prediction. For instance, it is known that older people tend to prefer music that was popular when they were in their early twenties [11]. This finding suggests that age is a useful variable to make a—very rough—prediction about the kind of music a person may have made use of in her or his adolescence. An individual’s personality traits may also help predict young people’s musical involvement [10]. Individuals who score high on sensation seeking, for instance, are more likely to actively seek music with a higher energy potential to attain their goals [12,13,14]. Despite such correlations, it would be extremely hard to identify and measure all the relevant exogenous variables and analyze how they might influence the kind of music a person is exposed to. And, again, some of these variables are just random and thus unpredictable. In consequence, a large part of the variance will remain unexplained. It appears that people can happen to fall in love with nearly every kind of music as long as they have positive experiences with it.

### Limitations of the Present Study

First, the research design of the present study was a compromise between parsimonious data collection and methodological rigor. The learning hypothesis presented in the Introduction actually calls for a longitudinal design, but that would have required surveying a sample of participants for many years. With the present design I tried to circumvent this obstacle by asking participants for the functional use of music in the past, aiming at measuring processes that occurred earlier in life. Nevertheless, this measure might be prone to recall bias because people who have a stronger MP at the time of the study may attribute more importance to the functions of music in the past. Second, as with most online studies, the present study was done with a convenient sample. There might be a bias toward participants who put a rather high value on music and are therefore more willing to participate in a survey like this. To reduce potential sampling bias, nonstudent populations, older people, and professional musicians were actively invited to participate in the study. Third, the instruction to pay attention to music-listening situations during the day might have altered participants’ usual behavior and experience of the situation. Specifically, they might have been much more mindful of the effects of music listening than they usually are. Fourth, there may be many more control variables that would be worth including in the estimation models. For instance, it might be that time of day or other environmental conditions have an influence on both the effects of music listening and the strength of MP.

### Conclusions

When people listen to music they usually try to attain specific goals. Those related to self-awareness and the regulation of mood and arousal are relatively important to them and those related to social relatedness less so. The same pattern is true for the strength of the effects that listening to music actually unleashes in the listener. Regardless of the specific effects that music evokes, the present results show that the more effective the music, the higher is the strength of MP listeners report. In addition, there is interindividual variance in both the strength of the effects music evokes and the strength of MP. The variance in both variables can be explained by listeners’ positive functional experiences with music in the past. Thus, the functional experiences people have with music throughout their lives may be the key to understanding the emergence and development of MP.

## Supporting Information

S1 Table Dataset of the study (Level 1 variables). (XLSX)

S2 Table Dataset of the study (Level 2 variables). (XLSX)
